# Disparate Associations of 24-h Central Aortic and Brachial Cuff Blood Pressure With Hypertension-Mediated Organ Damage and Cardiovascular Risk

**DOI:** 10.3389/fcvm.2022.795509

**Published:** 2022-02-22

**Authors:** Yueliang Hu, Jiehui Zhao, Qian Wang, Huijuan Chao, Biwen Tang, Di Cheng, Isabella Tan, Mark Butlin, Alberto Avolio, Weiliang Wang, Junli Zuo

**Affiliations:** ^1^Department of Geriatrics, Ruijin Hospital, Shanghai Jiao Tong University School of Medicine, Shanghai, China; ^2^Daning Community Health Service Center, Shanghai, China; ^3^Macquarie Medical School, Faculty of Medicine, Health and Human Sciences, Macquarie University, Sydney, NSW, Australia; ^4^Xuhui Center Hospital, Shanghai, China

**Keywords:** central blood pressure, peripheral blood pressure, atherosclerotic cardiovascular disease, hypertension-mediated organ damage, non-invasive haemodynamics indices

## Abstract

**Objective:**

Aim of this study was to evaluate the associations of non-invasive central aortic and peripheral (brachial) blood pressure (BP) for Hypertension-mediated organ damage (HMOD) and atherosclerotic cardiovascular disease (ASCVD) risk.

**Methods:**

We evaluated associations of HMOD with 24-h ambulatory blood pressure monitoring (ABPM) of central aortic and peripheral BP indices in patients with primary hypertension and presence of several cardiovascular risk factors. BP measurements were performed by means of a non-invasive automated oscillometric device (Mobil-O-Graph). HMOD was defined as the presence of carotid intima-media thickness (IMT) above normal values and/or carotid plaque, left ventricular hypertrophy (LVH), and/or renal abnormalities as assessed by urine albumin/creatinine ratio above normal values and/or estimated glomerular filtration rate (eGFR) <60 ml/min per 1.73 m^2^.

**Results:**

In the study cohort of 273 (age 55.2 ± 13.4 years, 71.8% male) patients with primary hypertension, documented HMOD was present in 180 (65.9%), LVH in 70 (25.6%), increased IMT in 129 (47.3%). Fifty-six patients (20.5%) had kidney organ damage (20.5% albuminuria and 2.6% impaired eGFR). When accounting for confounding factors (age, sex, body-mass-index, antihypertensive treatment, smoking, triacylglycerol, statin treatment, glucose, hypoglycemic therapy, or heart rate) only peripheral 24-h pulse pressure (PP) maintained statistical significance with HMOD indices (OR: 1.126, 95% CI: 1.012~1.253; *p* = 0.029). Using ASCVD risk score as the independent continuous variable in multiple linear regression, 24-h central systolic pressure (SBP) (β = 0.179; 95% CI:0.019~0.387; *p* = 0.031), daytime central PP (β = 0.114; 95% CI:0.070~0.375; *p* = 0.005, night-time central SBP (β = 0.411; 95% CI:0.112~0.691; *p* = 0.007) and night-time PP (β = 0.257; 95% CI:0.165~0.780; *p* = 0.003) were all positively associated with ASCVD risk.

**Conclusions:**

Blood pressure obtained by 24-h ABPM was better correlated with HMOD than office BP. Whilst 24-h peripheral BP showed a stronger association with HMOD than 24-h central BP, the prognostic value of 24-h central BP for the 10-year ASCVD risk was superior to 24-h peripheral BP.

## Introduction

With the change of lifestyle, the total number of hypertension patients in China has reached the alarming number of 245 million (1). As the most important risk factor for cardiovascular disease, hypertension has brought us a serious burden of disease. Statistics show that the prevalence and mortality of cardiovascular diseases in the Chinese are still on the rise. In 2017, 2.54 million people in China died due to high blood pressure (BP), of which 95.7% died of cardiovascular disease (2). Interventions for hypertension, especially early intervention, are urgently needed.

Changes in large arteries and microcirculation play a role in the mechanism of BP and HMOD (hypertension-mediated organ damage) (3). The increase in arterial stiffness caused by aging and other factors lead to an increase in systolic BP (SBP). The increase in SBP and pulse pressure (PP) transmit to the distal vascular system, leading to microcirculation damage, remodeling, and related endothelial dysfunction. Eventually, target organs such as heart, brain and kidney and blood vessels themselves are damaged.

HMOD is related to increased vascular risk and death, and their prevention should become the target of hypertension treatment and a surrogate sign of whether BP is well-controlled (4). It is theorized that at the time of diagnosis, all adult hypertension patients have HMOD, but the severity varies. Cardiovascular death, myocardial infarction, stroke, end-stage renal disease (ESRD) or heart failure are common and irreversible late complications (4). For young hypertensive patients, it is more important to prevent the development of HMOD.

The Chinese Hypertension Guidelines have pointed out that assessing HMOD is an important part of the diagnosis and evaluation of hypertension. After early detection and treatment, asymptomatic subclinical HMOD can be reversed, which helps to improve the prognosis of patients (5).

In this study, we investigated the use different indicators to evaluate the correlation between different BP indices and HMOD such as heart, kidney, and large blood vessel damage, hoping to clearly distinguish the intervention targets for early detection, intervention and reversal of HMOD.

## Materials and Methods

### Study Population

In this cross-sectional study between July 2018 and July 2020, 273 (196 men, age 55.21 ± 13.37 years) inpatients enrolled in the Department of Geriatrics, Shanghai Ruijin Hospital, China, who suffered from primary hypertension with presence of several cardiovascular risk factors or complications involving clinical HMOD were recruited. The inclusion criteria were 3 consecutive measurements of office SBP ≥140 mmHg or consultation diastolic BP (DBP) ≥90 mmHg after resting for 5 min in the supine position, or the use of antihypertensive drugs without the diagnosis of secondary hypertension. Exclusion criteria were clinical or laboratory evidence confirming the acute cardiocerebrovascular disease within the previous 3 months before enrollment or any life-threatening disease.

Patients' medical history, smoking habits, medication history, and auxiliary examinations [serum total cholesterol (TC), high-density lipoprotein cholesterol (HDL-C), low-density lipoprotein cholesterol (LDL-C), triglycerides (TG), blood glucose, serum creatinine, and urine albumin/creatinine ratio (ACR)] were collected in the form of standardized questionnaires. Cardiovascular risk was determined according to the American College of Cardiology/American Heart Association-Atherosclerotic Cardiovascular Disease (ACC/AHA-ASCVD) risk score (6).

Current smoking was defined as having smoked in their lifetime or smoked in the past 7 days of BP measurement (7, 8). Diabetes mellitus was defined as a fasting blood glucose concentration of at least 7 mmol/l, or a random blood glucose level of over 11.1 mmol/l, or the use of antidiabetic drugs. Dyslipidemia was defined as serum TC ≥240 mg/ dl, LDL-C ≥160 mg/dl, HDL-C ≤ 35 mg/dl, TG ≥150 mg/dl, or the use of antilipidemic drugs according to Adult Treatment Panel III cardiovascular risk factor definitions (9). The study protocol was reviewed and approved by the Ethics Committee of Ruijin Hospital, Shanghai Jiaotong University School of Medicine. All patients provided written informed consent.

### Blood Pressure Measurements

Both office and 24-h BP measurements were performed by means of a non-invasive automated oscillometric device (Mobil-O-Graph PWV; IEM, Stolberg, Germany), validated for brachial BP measurement, according to the European Society of Hypertension International protocol (10).

The monitor was fitted on a weekday between 8:00 am and 8:00 am. After 5-min rest, BP was measured four times consecutively at 1-min intervals. The mean of these measurements was settled as office(clinic) BP. The device was set to function under the manufacturer's inbuilt protocol number 11, namely: four BP recordings per hour from 0800 to 2,359 h and two recordings per hour from 0000 to 0759 h. In this mode of function, the device performs both brachial BP measurement and brachial pressure wave recording simultaneously. The aortic (central) BP is assessed by software analysis [with the application of validated generalized transfer functions (11, 12)] after the data are downloaded to the manufacturer's software (HMS version 4.6).The aortic BP derived by the Mobil-O-Graph NG apparatus has been compared vs. the non-invasive “gold standard” apparatus for central BP estimation (Sphygmocor device) in four studies (12–17), as well as vs. the invasive aortic BP recordings in one study (13), with consistently quite acceptable results (11, 14). The feasibility and/or reproducibility of the device to assess central hemodynamics, both in large populations at rest (17) and during ambulatory monitoring (15, 18), has been also evaluated with satisfactory results.

All patients included in the study had recordings of good technical quality (at least 80% of valid readings). Otherwise, ABPM was repeated in 1 week.

PPA was defined as the peripheral-to-central pulse pressure ratio and was calculated using the formula: PPA = (brachial SBP– brachial DBP)/(central SBP – central DBP).

### HMOD Indices

HMOD was defined as the presence of carotid intima-media thickness (IMT) above normal values (0.9 mm) and/or carotid plaque, left ventricular hypertrophy (LVH), and/or renal abnormalities as assessed by urine albumin/creatinine ratio (ACR) above normal values (>3.5 mg/mmol in females and >2.5 mg/mmol in males) and/or an estimated glomerular filtration rate (eGFR) <60 ml/min per 1.73 m^2^.

#### Intima-Media Thickness

We used high-resolution echocardiography Doppler ultrasound (HD11EX Ultrasound; Philips Medical Systems, Andover, MA, USA) with a broadband linear array transducer (multiple frequency: 4–12 MHz) to examine Carotid IMT.IMT was measured on both the left and right common carotid artery starting ~1.5 cm proximal to the carotid artery bulb. During the test, three records were collected from the left and right carotid arteries, and the average value of each side was calculated. When IMT> 1.3 mm, carotid plaque was diagnosed. When IMT ≥ 0.9 mm and/or carotid plaque was present, carotid artery abnormality was diagnosed.

#### Left Ventricular Hypertrophy

LVH was defined as a left ventricular mass index (LVMI) ≥ 115 g/m^2^ in men or ≥95 g/m^2^ in women and was calculated by means of echocardiography, performed according to the American Society of Echocardiography recommendations (19).

#### Renal Abnormalities

The definition and the diagnostic criteria for chronic kidney disease (CKD) were proposed in the Kidney Disease Outcomes Quality Initiative (K/DOQI) guidelines: (eGFR <60 ml/min/1.73 m^2^) as calculated by the MDRD formula (20) or urinary ACR > 3.5 mg/mmol in females and >2.5 mg/mmol in males were used to screen hypertensive patients with CKD.

### The Atherosclerotic Cardiovascular Disease (ASCVD) Risk

The ACC/AHA-ASCVD risk score was determined using values of TC, HDL-C, LDL-C, age, BP, gender, presence of diabetes, and smoking status (6). The ACC/AHA-ASCVD risk score was defined as high risk when the score ≥7.5% and low risk when score <7.5% (21).

### Statistical Analysis

We use SPSS 24.0 for Windows (SPSS Inc., Chicago, IL, USA) for statistical analysis. When *p* < 0.05 on both sides, it is considered statistically significant.

Continuous variables are expressed as mean ± SD, and frequencies (percentage) are reported for categorical variables. Student's *t*-test or Chi-square test were used for the comparison between patients with and without HMOD. Pearson correlation was used to assess the relations between brachial, central aortic BP and LVMI, ACR, eGFR, or IMT. The association of each BP estimate with the presence of HMOD was assessed by means of logistic regression analyses, with odds ratio (OD) calculation adjusted for age, sex, body mass index (BMI), antihypertensive treatment, smoking, TG, statin treatment, fasting glucose, glucose, hypoglycemic therapy, heart rate.

Furthermore, the relative impact of central vs. peripheral BP estimates was assessed by the simultaneous introduction of each pair of central and peripheral measurements (office, 24-h, daytime, and night-time) in the logistic regression models, and then calculating the adjusted ODs. The association of each BP estimate with the presence of ASCVD risk was assessed by means of linear regression with adjustment for baseline covariates including age, sex, BMI, antihypertensive treatment, smoking, TG, statin treatment, fasting glucose, glucose, hypoglycemic therapy, heart rate.

## Results

### Characteristics of Participants

The study cohort comprised 273 (71.8% men) patients with primary hypertension. A total of 180 (65.9%) patients had documented HMOD. LVH was present in 70 patients (25.6%), increased IMT in 129 (47.3%). A total of 56 patients (20.5%) had kidney organ damage (20.5% albuminuria and 2.6% impaired eGFR). From them, 66 patients (24.2%) presented HMOD in more than one organ.

Table 1 shows the difference in clinical parameters and BP measurements between patients with and without HMOD. Patients with HMOD had significantly higher brachial and aortic (central) BP, measured during 24-h, day or night except clinic peripheral PP and clinic aortic PP. Patients with HMOD were older, more frequently treated, and more with associated glucose disorders. As expected, values of serum creatinine, ACR, LVMI, IMT, and ASCVD risk score were increased and eGFR reduced in the group with HMOD. The PPA was higher in HMOD group, but there was no statistical significance.

**Table 1:** Characteristics of the study subjects.

**Characteristics**	**Total**	**Without HMOD**	**With HMOD**	***P-*value**
	**(*n* = 273)**	**(*n* = 93)**	**(*n* = 180)**	
Age,y	55.21 ± 13.37	50.10 ± 12.59	57.82 ± 13.02	<0.001
Sex (% men)	28.2	28.9	26.9	0.727
BMI/(kg/m^2^)	26.49 ± 5.09	26.32 ± 5.53	26.57 ± 4.87	0.701
Antihypertensive treatment (%)	83.5	75.3	87.8	0.008
Glucose (mmol/L)	5.92 ± 1.59	5.5 ± 1.10	6.14 ± 1.76	0.002
Triglycerides (mmol/L)	2.06 ± 1.97	2.09 ± 1.29	2.04 ± 2.24	0.854
Total cholesterol (mmol/L)	4.73 ± 0.98	4.80 ± 0.78	4.69 ± 1.07	0.384
LDL-C (mmol/L)	3.13 ± 0.75	3.19 ± 0.60	3.09 ± 0.82	0.320
Creatinine (μmol/L)	78.59 ± 15.50	77.10 ± 12.11	79.36 ± 16.96	0.256
ASCVD(%)	15.00 ± 13.07	9.54 ± 8.63	17.60 ± 14.01	<0.001
eGFR(ml/min per 1.73 m^2^)	90.24 ± 15.53	93.19 ± 13.50	88.71 ± 16.31	0.024
LVMI (g/m^2^)	98.18 ± 23.09	86.47 ± 13.68	104.23 ± 24.62	<0.001
IMT (mm)	0.73 ± 0.10	0.69 ± 0.07	0.75 ± 0.11	<0.001
LgACR (mg/mmol)	0.43 ± 0.45	0.25 ± 0.24	0.53 ± 0.50	<0.001
Clinic Peripheral SBP	133.17 ± 18.45	129.76 ± 18.01	134.94 ± 18.44	0.029
Clinic Aortic SBP	123.81 ± 16.90	120.78 ± 15.60	125.38 ± 17.37	0.044
Clinic Peripheral PP	44.86 ± 13.39	42.72 ± 12.93	45.97 ± 13.52	0.059
Clinic Aortic PP	33.72 ± 10.90	32.41 ± 10.51	34.41 ± 11.07	0.176
Clinic PPA	1.32 ± 0.17	1.31 ± 0.13	1.33 ± 0.19	0.380
24-h Peripheral SBP	126.69 ± 11.94	122.76 ± 11.41	128.74 ± 11.72	<0.001
24-h Aortic SBP	118.11 ± 11.05	115.46 ± 11.30	119.51 ± 10.69	0.004
24-h Peripheral PP	43.00 ± 9.27	40.35 ± 7.79	44.38 ± 9.70	0.001
24-h Aortic PP	33.07 ± 7.09	31.87 ± 7.00	33.71 ± 7.08	0.043
24-h PPA	1.31 ± 0.12	1.29 ± 0.07	1.32 ± 0.15	0.051
Daytime Peripheral SBP	128.06 ± 12.06	124.41 ± 12.03	129.97 ± 11.67	<0.001
Daytime Aortic SBP	119.09 ± 11.07	116.17 ± 10.84	120.62 ± 10.90	0.002
Daytime Peripheral PP	42.79 ± 9.05	40.58 ± 8.02	43.94 ± 9.35	0.004
Daytime Aortic PP	32.33 ± 6.86	30.94 ± 6.00	33.07 ± 7.18	0.015
Daytime PPA	1.33 ± 0.10	1.31 ± 0.08	1.34 ± 0.10	0.081
Night-time Peripheral SBP	122.95 ± 14.52	117.66 ± 12.27	125.66 ± 14.86	<0.001
Night-time Aortic SBP	115.43 ± 13.12	111.05 ± 11.32	117.67 ± 13.44	<0.001
Night-time Peripheral PP	42.86 ± 9.92	39.93 ± 8.57	44.35 ± 10.25	<0.001
Night-time Aortic PP	34.20 ± 7.34	32.4 ± 7.03	35.14 ± 7.35	0.004
Night-time PPA	1.25 ± 0.12	1.24 ± 0.09	1.26 ± 0.13	0.127

*Data are mean ± SD or percentage as marked. P-value: independent t-test analysis of variance for numeric variables and chi-square test for categoric variables*.

*BMI, body mass index; LDL-C, low density lipoprotein cholesterol; SBP, systolic blood pressure; PP, pulse pressure; ASCVD, atherosclerotic cardiovascular disease; eGFR, an estimate of GFR for the modified MDRD formula; ACR, Urine albumin-to-creatinine; PPA, peripheral-to-central pulse pressure ratio*.

Table 2 summarizes the correlations between peripheral and central BP and HMOD indices. There were different strengths of correlation between BP and HMOD indices. LVMI was significantly associated with both brachial and central SBP parameters for clinic, 24-h, day and night (*r* = 0.121–0.281, *p* < 0.001). ACR was significantly associated with both brachial and central SBP as well as PP parameters for clinic,24-h, day and night (*r* = 0.151–0.299, *p* < 0.01) except clinic aortic SBP and clinic aortic PP. It was also significantly associated with Clinic PPA and Night-time PPA (*r* = 0.147–0.148, *p* < 0.05) except 24-h PPA and Daytime PPA.There was a significant negative association between eGFR and the brachial and central PP for 24-h, day and night (*r* = −0.205 to −0.234, *p* < 0.001) except night-time aortic PP. It was also significantly associated with Clinic PPA and Night-time PPA (*r* = −0.134 to −0.144, *p* < 0.05) except 24-h PPA and Daytime PPA.IMT was significantly associated with both brachial and central PP parameters for 24-h, day and night (*r* = 0.141–0.163, *p* < 0.05).

**Table 2:** Relationship between BP and HMOD.

**Variable**	**LVMI**		**logACR**		**eGFR**		**IMT**	
	***r-*value**	***P-*value**	***r* value**	***P-*value**	***r-*value**	***P-*value**	***r-*value**	***P-*value**
Age,y	0.065	0.287	0.109	0.072	−0.404^**^	<0.001	0.350^**^	<0.001
Sex	−0.225^**^	<0.001	0.096	0.113	−0.176^**^	0.004	−0.079	0.208
BMI/(kg/m^2^)	0.105	0.086	−0.070	0.251	0.086	0.158	0.037	0.554
Glucose (mmol/L)	0.070	0.264	0.286^**^	<0.001	−0.060	0.341	0.125	0.052
Triglyceride (mmol/L)	0.194^**^	0.001	0.020	0.737	−0.021	0.736	−0.213^**^	0.001
Total cholesterol (mmol/L)	−0.165^**^	0.006	0.006	0.925	−0.121^*^	0.047	−0.061	0.331
LDL–C (mmol/L)	−0.157^**^	0.010	0.017	0.780	−0.113	0.063	−0.045	0.475
Clinic Peripheral SBP	0.236^**^	<0.001	0.161^*^	0.008	0.024	0.700	0.079	0.212
Clinic Aortic SBP	0.255^**^	<0.001	0.111	0.083	0.071	0.271	0.048	0.472
Clinic Peripheral PP	0.111	0.069	0.161^**^	0.008	−0.113	0.064	0.101	0.108
Clinic Aortic PP	0.088	0.173	0.066	0.308	−0.063	0.329	0.079	0.233
Clinic PPA	0.115	0.073	0.147^*^	0.022	−0.134^*^	0.037^*^	0.049	0.461
24–h Peripheral SBP	0.281^**^	<0.001	0.299^**^	<0.001	−0.058	0.343	0.096	0.127
24–h Aortic SBP	0.252^**^	<0.001	0.151^*^	0.013	0.005	0.939	0.110	0.081
24–h Peripheral PP	0.100	0.100	0.286^**^	<0.001	−0.234^**^	<0.001	0.157^*^	0.012
24–h Aortic PP	0.079	0.194	0.237^**^	<0.001	−0.205^**^	0.001	0.163^**^	0.009
24–h PPA	0.073	0.230	0.043	0.477	−0.060	0.328	0.042	0.506
Daytime Peripheral SBP	0.265^**^	<0.001	0.280^**^	<0.001	−0.047	0.439	0.085	0.176
Daytime Aortic SBP	0.254^**^	<0.001	0.232^**^	<0.001	0.006	0.924	0.064	0.312
Daytime Peripheral PP	0.077	0.208	0.279^**^	<0.001	−0.229^**^	<0.001	0.141^*^	0.025
Daytime Aortic PP	0.056	0.363	0.258^**^	<0.001	−0.225^**^	<0.001	0.153^*^	0.015
Daytime PPA	0.074	0.223	0.062	0.308	−0.004	0.954	−0.027	0.664
Night–time Peripheral SBP	0.253^**^	<0.001	0.297^**^	<0.001	−0.075	0.220	0.109	0.082
Night–time Aortic SBP	0.255^**^	<0.001	0.250^**^	<0.001	−0.015	0.808	0.099	0.118
Night–time Peripheral PP	0.121^*^	0.048	0.277^**^	<0.001	−0.208^**^	0.001	0.148^*^	0.018
Night–time Aortic PP	0.078	0.202	0.155^*^	0.011	−0.111	0.072	0.154^*^	0.014
Night-time PPA	0.033	0.596	0.148^*^	0.016	−0.144^*^	0.019	−0.014	0.827

* *P < 0.05;*

** *P < 0.01. BMI, body mass index; LDL-C, low density lipoprotein cholesterol; SBP, systolic blood pressure; PP, pulse pressure; eGFR, is an estimate of GFR for the modified MDRD formula*.

**Figure 1:**
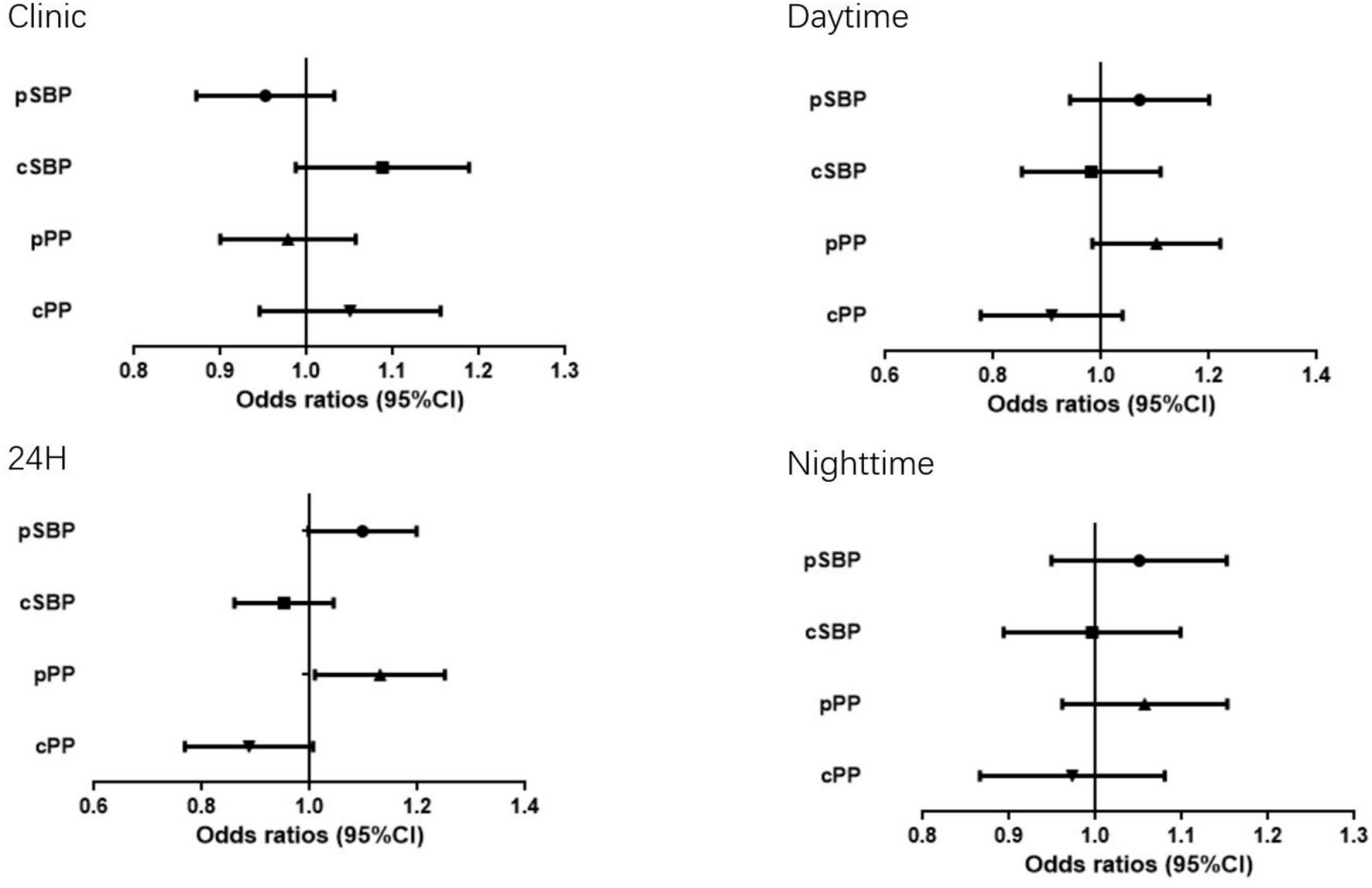
Odds ratio (OR) and 95% Confidence Interval (CI) of Hypertension-mediated organ damage with the all BP measurements.

Table 3 shows adjusted ODs for office and ambulatory BP estimates. When each pair of BP value (central and peripheral) were introduced in the same logistic regression model, only peripheral BP estimates (24-h pPP) maintained their statistical significance, whereas none of the central BP parameters were significant after adjustment for age, sex, BMI, antihypertensive treatment, smoking, TG, statin treatment, glucose, hypoglycemic therapy, heart rate (Figure 1).

**Table 3:** Odds ratios and 95% confidence intervals for each mmHg increase of the association of each blood pressure value with the presence of Hypertension-mediated organ damage.

	**Model 1**	**Model 2**	**Model 3**	**Model 4**	**Model 5**	**Model 6**
Clinic Peripheral SBP	1 (0.935~1.07)	0.995 (0.929~1.065)	0.997 (0.931~1.067)	0.999 (0.933~1.07)	1 (0.929~1.076)	0.950 (0.873~1.033)
Clinic Aortic SBP	1.025 (0.952~1.103)	1.028 (0.954~1.108)	1.026 (0.952~1.105)	1.024 (0.95~1.103)	1.026 (0.947~1.112)	1.085 (0.989~1.190)
Clinic Peripheral PP	1.022 (0.961~1.086)	1.012 (0.951~1.077)	1.014 (0.953~1.079)	1.016 (0.954~1.082)	1.02 (0.955~1.091)	0.976 (0.901~1.058)
Clinic Aortic PP	0.985 (0.915~1.06)	0.996 (0.924~1.073)	0.995 (0.923~1.073)	0.993 (0.921~1.071)	0.993 (0.916~1.076)	1.047 (0.947~1.157)
24-h pSBP	***1.095 (1.013** **~** **1.184)***	***1.092 (1.01** **~** **1.181)***	***1.092 (1.01** **~** **1.182)***	***1.092 (1.009** **~** **1.182)***	***1.11 (1.011** **~** **1.218)***	1.095 (0.999~1.200)
24-h cSBP	0.951 (0.876~1.033)	0.95 (0.874~1.032)	0.949 (0.873~1.032)	0.95 (0.873~1.033)	0.937 (0.849~1.035)	0.949 (0.862~1.046)
24-h pPP	***1.121 (1.017** **~** **1.235)***	***1.114 (1.015** **~** **1.223)***	***1.115 (1.015** **~** **1.224)***	***1.116 (1.016** **~** **1.227)***	***1.136 (1.026** **~** **1.257)***	**1.126 (1.012** **~** **1.253)**
24-h cPP	0.894 (0.791~1.01)	0.897 (0.798~1.009)	0.897 (0.798~1.009)	0.896 (0.796~1.009)	0.885 (0.778~1.006)	0.882 (0.771~1.009)
Daytime pSBP	1.073 (0.964~1.195)	1.068 (0.959~1.188)	1.068 (0.959~1.188)	1.067 (0.958~1.189)	1.086 (0.97~1.215)	1.066 (0.945~1.203)
Daytime cSBP	0.97 (0.864~1.09)	0.972 (0.865~1.091)	0.971 (0.865~1.091)	0.972 (0.865~1.093)	0.958 (0.848~1.082)	0.976 (0.856~1.113)
Daytime pPP	***1.11 (1.009** **~** **1.222)***	***1.104 (1.003** **~** **1.215)***	***1.104 (1.004** **~** **1.215)***	***1.108 (1.005** **~** **1.22)***	***1.12 (1.013** **~** **1.239)***	1.098 (0.986~1.223)
Daytime cPP	0.896 (0.791~1.015)	0.9 (0.794~1.019)	0.9 (0.794~1.019)	0.896 (0.79~1.017)	0.888 (0.777~1.015)	0.902 (0.780~1.043)
Night-time pSBP	1.021 (0.936~1.114)	1.022 (0.937~1.116)	1.023 (0.937~1.117)	1.025 (0.938~1.12)	1.053 (0.962~1.153)	1.047 (0.951~1.154)
Night-time cSBP	1.024 (0.932~1.125)	1.019 (0.927~1.12)	1.018 (0.926~1.12)	1.016 (0.924~1.118)	0.991 (0.9~1.092)	0.993 (0.895~1.100)
Night-time pPP	1.042 (0.96~1.131)	1.038 (0.957~1.127)	1.039 (0.957~1.127)	1.041 (0.958~1.13)	1.067 (0.981~1.16)	1.054 (0.963~1.154)
Night-time cPP	0.985 (0.89~1.09)	0.987 (0.893~1.092)	0.987 (0.892 ~1.092)	0.985 (0.89~1.09)	0.963 (0.869~1.066)	0.969 (0.868~1.082)

*pSBP, Peripheral systolic blood pressure; cSBP, Aortic systolic blood pressure; pPP, Peripheral pulse pressure; cPP, Aortic pulse pressure*.

*Model 1 adjusting for Age,Sex, BMI*.

*Model 2 adjusting for Age,Sex, BMI, Antihypertensive treatment*.

*Model3 adjusting for Age, Sex, BMI, Antihypertensive treatment ,Smoker*.

*Model 4 adjusting for Age, Sex, BMI, Antihypertensive treatment,Smoker, Triacylglycerol*.

*Model 5 adjusting for Age, Sex, BMI, Antihypertensive treatment,Smoker, Triacylglycerol, Statin treatment*.

*Model 6 adjusting for Age, Sex, BMI, Antihypertensive treatment, Smoker, Triacylglycerol, Statin treatment, Glucose, Hypoglycemic therapy, HR*.

*The bold values represent the values that are statistically significant (P < 0.05)*.

When PPA (Clinic, 24-h PPA, Daytime, Night-time) were introduced in the same logistic regression model, none of PPA parameters were significant after adjustment for age, sex, BMI, antihypertensive treatment, smoking, TG, statin treatment, glucose, hypoglycemic therapy, heart rate.

The left ventricular mass was further used as an independent HMOT index and analyzed as the independent continuous variable in multiple linear regression, we found that 24-h peripheral SBP was more relevant to LVMI than 24-h central SBP (β = 0.161; 95% CI:0.064~0.562; *p* = 0.014) after adjustment for age, sex, BMI, antihypertensive treatment, smoking, TG, statin treatment, glucose, hypoglycemic therapy, heart rate. Night-time central SBP is more relevant to LVMI than night-time peripheral SBP (β = 0.134; 95% CI:0.008~0.473; *p* = 0.042) when accounting for confounding factors, Using ASCVD risk score as the independent continuous variable in multiple linear regression, central but not peripheral 24-h SBP, daytime PP, night-time SBP and PP were all positively associated with ASCVD risk after adjusted for traditional cardiovascular risk factors (Table 4). In addition, Daytime PPA(β = −0.096; 95% CI:−0.238 to −0.036.387; *p* = 0.008) and Night-time PPA (β = −0.098; 95% CI:−0.193 to −0.030; *p* = 0.007)were all negatively ssociated with ASCVD risk after adjusted for confounding factors except Clinic PPA and 24-h PPA.

**Table 4:** Linear regression analysis of associations between each blood pressure value and ASCVD.

		**ASCVD (%)**			
**Variable**	**B**	**SE**	**β**	***P-*value**	**95% CI**
**Model 1**					
24-h pSBP (mmHg)	0.167	0.166	0.153	0.152	−0.062~0.395
24-h cSBP (mmHg)	0.158	0.124	0.136	0.204	−0.087~0.403
24-h pPP	0.047	0.121	0.032	0.701	−0.192~0.285
24–h cPP	0.341	0.149	0.182	0.023^*^	0.048~0.634
Daytime pSBP	−0.05	0.231	−0.046	0.83	−0.506~0.406
Daytime cSBP	0.376	0.25	0.321	0.135	−0.117~0.87
Daytime pPP	−0.141	0.207	−0.092	0.498	−0.549~0.267
Daytime cPP	0.592	0.266	0.3	0.027^*^	0.066~1.117
Night-time pSBP	−0.148	0.171	−0.167	0.386	−0.485~0.188
Night–time cSBP	0.43	0.185	0.437	0.021^*^	0.065~0.796
Night-time pPP	−0.053	0.16	−0.038	0.741	−0.368~0.263
Night-time cPP	0.393	0.2	0.216	0.051	−0.001~0.786
**Model 2**					
24-h pSBP (mmHg)	0.141	0.113	0.13	0.214	−0.082~0.365
24-h cSBP (mmHg)	0.139	0.122	0.119	0.254	−0.101~0.378
24-h pPP	0.082	0.116	0.056	0.483	−0.148~0.311
24–h cPP	0.258	0.144	0.138	0.074	−0.025~0.542
Daytime pSBP	−0.072	0.226	−0.067	0.75	−0.516~0.373
Daytime cSBP	0.356	0.244	0.304	0.146	−0.125~0.837
Daytime pPP	−0.146	0.199	−0.096	0.465	−0.538~0.246
Daytime cPP	0.556	0.256	0.282	0.031^*^	0.051~1.06
Night–time pSBP	−0.145	0.167	−0.163	0.388	−0.475~0.185
Night-time cSBP	0.384	0.182	0.39	0.036^*^	0.026~0.743
Night–time pPP	−0.086	0.154	−0.062	0.578	−0.389~0.218
Night–time cPP	0.376	0.192	0.207	0.051	−0.002~0.754
**Model 3**					
24–h pSBP (mmHg)	0.127	0.104	0.117	0.224	−0.078~0.331
24–h cSBP (mmHg)	0.138	0.111	0.119	0.215	−0.081~0.358
24–h pPP	0.07	0.106	0.048	0.509	−0.139~0.279
24-h cPP	0.296	0.131	0.158	0.025^*^	0.038~0.555
Daytime pSBP	−0.155	0.207	−0.143	0.454	−0.562~0.252
Daytime cSBP	0.431	0.224	0.367	0.055	−0.01~0.871
Daytime pPP	−0.208	0.181	−0.137	0.252	−0.566~0.149
Daytime cPP	0.641	0.233	0.326	0.006^*^	0.181~1.101
Night–time pSBP	−0.092	0.155	−0.104	0.551	−0.397~0.212
Night–time cSBP	0.309	0.168	0.314	0.068	−0.023~0.64
Night-time pPP	−0.078	0.14	−0.056	0.575	−0.354~0.197
Night-time cPP	0.374	0.174	0.206	0.033^*^	0.031~0.718
**Model4**					
24-h pSBP (mmHg)	0.127	0.104	0.117	0.226	−0.079~0.332
24-h cSBP (mmHg)	0.137	0.112	0.118	0.221	−0.083~0.357
24-h pPP	0.067	0.106	0.046	0.527	−0.142~0.276
24-h cPP	0.299	0.131	0.16	0.024^*^	0.041~0.558
Daytime pSBP	−0.162	0.207	−0.15	0.437	−0.57~0.247
Daytime cSBP	0.436	0.224	0.372	0.053	−0.006~0.878
Daytime pPP	−0.236	0.183	−0.155	0.198	−0.596~0.124
Daytime cPP	0.676	0.235	0.344	0.004^*^	0.213~1.14
Night-time pSBP	−0.102	0.156	−0.115	0.512	−0.409~0.204
Night-time cSBP	0.316	0.169	0.321	0.062	−0.017~0.649
Night–time pPP	−0.084	0.14	−0.061	0.55	−0.361~0.193
Night-time cPP	0.379	0.175	0.208	0.031^*^	0.035~0.723
**Model 5**					
24-h pSBP (mmHg)	0.127	0.104	0.117	0.225	−0.078~0.332
24-h cSBP (mmHg)	0.135	0.112	0.116	0.227	−0.085~0.356
24-h pPP	0.053	0.106	0.036	0.617	−0.157~0.263
24-h cPP	0.315	0.132	0.168	0.018^*^	0.055~0.574
Daytime pSBP	−0.166	0.207	−0.154	0.423	−0.575~0.242
Daytime cSBP	0.44	0.224	0.375	0.051	−0.002~0.881
Daytime pPP	−0.246	0.183	−0.161	0.18	−0.605~0.114
Daytime cPP	0.688	0.235	0.349	0.004^*^	0.225~1.151
Night-time pSBP	−0.087	0.157	−0.098	0.579	−0.397~0.222
Night-time cSBP	0.299	0.17	0.304	0.081	−0.037~0.635
Night-time pPP	−0.074	0.142	−0.053	0.601	−0.354~0.205
Night-time cPP	0.366	0.177	0.201	0.039^*^	0.018~0.714
**Model 6**					
24–h pSBP (mmHg)	−0.008	0.088	−0.007	0.931	−0.181~0.166
24–h cSBP (mmHg)	0.203	0.093	0.179	0.031^*^	0.019~0.387
24-h pPP	0.026	0.088	0.018	0.771	−0.148~0.199
24-h cPP	0.15	0.11	0.081	0.175	−0.067~0.366
Daytime pSBP	−0.349	0.17	−0.334	0.042	−0.685~-0.013
Daytime cSBP	0.572	0.184	0.506	0.002^*^	0.209~0.936
Daytime pPP	0.107	0.06	0.071	0.076	−0.011~0.226
Daytime cPP	0.222	0.077	0.114	0.005^*^	0.070~0.375
Night-time pSBP	−0.263	0.139	−0.294	0.059	−0.537~0.010
Night-time cSBP	0.402	0.147	0.411	0.007^*^	0.112~0.691
Night-time pPP	−0.241	0.13	−0.170	0.064	−0.497~0.015
Night-time cPP	0.472	0.156	0.257	0.003^*^	0.165~0.780

*pSBP, Peripheral systolic blood pressure; cSBP, Aortic systolic blood pressure; pPP, Peripheral pulse pressure; cPP, Aortic pulse pressure*.

*Model 1 adjusting for Age, Sex, BMI*.

*Model 2 adjusting for Age, Sex, BMI, Antihypertensive treatment*.

*Model 3 adjusting for Age, Sex, BMI, Antihypertensive treatment, Smoker*.

*Model 4 adjusting for Age, Sex, BMI, Antihypertensive treatment, Smoker, Triacylglycerol*.

*Model 5 adjusting for Age, Sex, BMI, Antihypertensive treatment, Smoker, Triacylglycerol, Statin treatment*.

*Model 6 adjusting for Age, Sex, BMI, Antihypertensive treatment, Smoker, Triacylglycerol, Statin treatment, Glucose, Hypoglycemic therapy, HR*.

*The symbol*

* *and bold values represent the values that are statistically significant (P < 0.05)*.

## Discussion

ABPM data of HMOD patients showed significantly higher BP compared to patients without HMOD, but the clinic (office) BP did not increase significantly. This suggested that even for patients with normal clinic BP, we need to be vigilant and realize that clinic BP does not provide complete information.

Although HMOD and non-HMOD patients had no difference in clinic peripheral and central PP and SBP, HMOD patients showed significantly higher BP levels on multiple indicators during ABPM. Furthermore, only central but not peripheral BP was associated with increased 10-year ASCVD risk.

BP measurement is a fundamental means and method for evaluating BP levels, diagnosing hypertension, and observing the efficacy of antihypertensive treatment. In clinical and population prevention work, the main use of office BP measurement and out-of-office BP measurement, the latter includes ABPM and home BP monitoring (HBPM), can provide a large amount of BP data outside the medical environment (5).

Clinic BP, recommend by most clinical guidelines (22), has been considered as the basis for guiding therapeutic decisions focused on the cardiovascular protection (23), closely related to prognosis. However, in the last two decades, ABPM, developed initially to study the circadian changes in BP and to determine the influence of BP-lowering drugs on the 24-h BP profile (24), has become important in determining BP of individuals, as it provides a more accurate prognosis and a better association with HMOD in hypertensive patients with respect to clinic BP (25–28). In our study, compared to clinic BP, BP obtained during 24-h ABPM had a stronger correlation with HMOD, which suggests it could be routinely used for assessment of hypertensive patients with suspected HMOD.

We found that different BP indicators have different correlations with HMOD: SBP and PP are better correlated with ACR, PP is better correlated with eGFR and IMT, and SBP is better correlated with LVMI.

The increase of SBP will increase the vascular pressure at the end of systole, and the increase of PP will cause vascular endothelial damage and mechanical fatigue, leading to atherosclerosis. Kong MG et al. showed SBP and PP are better HMOD indicators (29).It showed patients undergoing elective invasive coronary angiography (CAG), SBP, and PP had stronger relationships with E/e' and coronary artery disease than DBP and mean arterial pressure (MAP).

Jokiniitty et al. (30) suggested that PP is the most significant BP indicator in predicting future LVMI and change in LVMI. Cirillo et al. (31) suggested that in non-diabetic, middle-aged adults, PP and isolated systolic hypertension are directly related to microalbuminuria. The South Korean study (29) suggested that neither SBP nor PP was related to LVMI and eGFR. A study by Chinese investigators (32) suggested that SBP and severe hypertension (grade 3) were common independent risk factors for HMOD. These differences may be related to sample size, BP level, and age of study cohort.

In general, PP is an independent risk factor for cardiovascular disease and mortality, and there is a clear link between PP and HMOD (29). In addition, the influence of SBP and overall BP levels cannot be ignored.

It is well-known that SBP and PP are higher when assessed at the brachial artery compared to the aorta because of PP amplification across the arterial tree (33). Therefore, target organs, especially the heart and large arteries, which are directly exposed to central rather than brachial BP, could be more strongly affected by the former (33). In fact, central BP has been increasingly considered to be a better estimator than the traditional peripheral BP measurement, found to be more closely related with HMOD, such as LV mass, carotid intima-media thickening, and pulse wave velocity (34). What is more, as shown by some longitudinal studies, central BP has a better predictive value of future cardiovascular events and mortality in hypertensive patients (35–38).

In most of the studies showing better predictive value of central BP, central and peripheral BP estimates were taken at clinic measurements of BP. In this study we investigated if ABPM might give the same result: whether it be assumed that 24-h central arterial pressure is superior to peripheral arterial pressure in predicting HMOD and long-term prognosis. However, seemingly contradictory results have been obtained in this study. The 24-h central BP did indeed show a greater predictive value for the 10-year ASCVD risk than 24-h peripheral BP. But when it comes to correlation with HMOD, the opposite is true: 24-h peripheral BP showed a stronger association with HMOD than 24-h central BP.This result is consistent with the findings of Spanish investigators (39).

The conclusion appears paradoxical. Why is the central ABPM better than the peripheral in 10-year CV risk but poorer in HMODs? In fact, in the past few years, research in this area has consistently shown conflicting results. Studies have suggested that the correlation between central BP and cardiovascular events is better than peripheral BP (35, 36). Other studies, like ours, have not found the advantage of central BP (37, 38) specific to HMOD, but rather for ASCVD. The difference in measurement and correction methods may be the reason for the different conclusions reached in different studies. In addition, we think the more critical point is that patients were mostly treated and this might have significantly influenced the results. After early detection and timely treatment, subclinical HMOD can be reversed (40). In contrast, the adverse association between cardiovascular outcomes and aortic sclerosis cannot be reversed by antihypertensive therapy, which may be one of the apparent reasons for the contradictory results of this study.

The finding of a lack of association with 24 h brachial BP and IMT is surprising and differs from several other findings in the general population (41) or in hypertensive patients (42). We postulate that the difference may be related to the following factors. Most of the selected population received antihypertensive therapy in our study, while the patients in the other two studies had no history of hypertension or were in the drug washout period. The effect of antihypertensive medication on patients' SBP and PP may be the main reason for the difference. In addition, the measurement method of IMT is different. Our IMT measurement is only for one carotid site, while the other two studies carried out 6~12 carotid sites. Further research in a larger sample size of the population may provide further clarification.

Our research results also show that if LVMI is analyzed as a stand-alone HMOD, 24-h peripheral SBP is more relevant to LVMI than 24-h central SBP. Night-time central SBP is more relevant to LVMI than night-time peripheral SBP. This is also contrary to what is generally accepted. It is generally believed that, compared with peripheral blood pressure, central BP is more closely correlated with LVMI (43). The paradox found in our study is most likely related to changes in antihypertensive treatment and arterial stiffness. Central SBP and PWV parameters are significantly correlated to hypertension (43). To address this issue, we are planning to analyze different PWV phenotypes in patients with hypertension.

Our study also showed that the clinic PPA, 24-h 24PPA, day PPA, night-time PPA had no prognostic value for HMOD. As the day PPA increased, the ASCVD risk decreased, while the Night-time PPA increased, the ASCVD risk decreased. 24-h PPA and clinic PPA had no prognostic value for ASCVD risk. In other words, PPA only has a prognostic effect on ASCVD risk, and has no correlation with HMOD. The Northern Shanghai Study (44) showed that PPA is a cardiac-related biomarker in community-based elderly. It seems understandable that, PPA is an indicator of cardiac pressure load, and it is related to cfPWV. As a vascular biomarker, cfPWV reflects the vessel elasticity of the aorta and is significantly associated with arterial changes, including glomerulus damage (44). PPA is only related to cardiac HMOD, so PPA and cfPWV have different prognostic values for HMOD, but they have similar values in ASCVD risk.

Due to the hypervagal function, night-time SBP decreased significantly (> 10%) so the day time peripheral PP was slightly higher than the night-time peripheral PP. Since the PPA of night time is less than the day time, day time SBP does not decrease significantly, resulting in a slightly higher night-time central PP than the day time central PP. Since the relation between central and peripheral PP is highly dependent on heart rate, is expected that changes in heart rate would result in different degree of amplification of PP from the aorta to the periphery.

In future research, we will address the PPA effect of ABPM at different times, especially the prognostic value of nighttime changes to the risk of HMOD and ASCVD.

In this study, HMOD was defined as the presence of carotid IMT above normal values (0.9 mm), LVH, and/or renal abnormalities as assessed by urine ACR above normal values (>3.5 mg/mmol in females and >2.5 mg/mmol in males) and/or an eGFR <60 ml/min per 1.73 m^2^.

Carotid artery plaque was classified as abnormal carotid artery in this study, and ABI and PWV measurements were not considered to be a manifestation of target organ damage. We adopt this definition of target organ damage to refer to the definition in the 2018 Chinese guidelines for the management of hypertension (40). It was a relatively simple and convenient method for detecting target organ damage. The predictive effect of ABI and PWV on patients' cardiovascular risk has been reported in many studies, but at present in China, it is still an investigative indicator rather than a clinical indicator, so we did not include these two indicators for analysis. We will conduct further analysis in future research.

### Limitations

This study has some limitations. As a cross-sectional study with a small sample size, the results need to be further confirmed in prospective studies. The cohort of this study is a physical examination population and the risk factors such as antihypertensive treatment werenot collected, therefore it is unknown whether results are applicable for other disease populations. The study was conducted in an Asian population, and it is not known whether the results will hold true for other ethnic groups.

## Conclusion

Blood pressure obtained by 24-h peripheral ABPM was better correlated with HMOD than office BP and 24-h central BP. However, the prognostic value of 24-h central BP for the 10-year ASCVD risk was superior to 24-h peripheral BP.

## Data Availability Statement

The data that support the findings of this study are available from the corresponding author, [Jl Z], upon reasonable request.

## Ethics Statement

Written informed consent was obtained from the individual(s) for the publication of any potentially identifiable images or data included in this article.

## Author Contributions

YH contributed to the concept and design of the study, the analysis and interpretation of data, and drafting and critically revising the manuscript. QW, JZ, HC, and BT were responsible for study design and analysis and interpretation of data. DC was responsible for the figure. IT contributed to interpretation of data and critically revising the manuscript. MB and AA contributed to the study design, the thorough reading of the manuscript and several revisions. JlZ and WW contributed to the conception and design of the study, interpretation of the data, and critically revising the manuscript. All authors approved the final version for publication and agree to be accountable for all aspects of the work in ensuring that questions related to the accuracy or integrity of any part of the work are appropriately investigated and resolved.

## Funding

Project Supported by the National Natural Science Foundation of China (Grant No. 81500190), and Clinical Science and Shanghai municipal hospital new frontier technology joint project (SHDC12019X20), Shanghai Municipal Commission of Health and Family Planning (Grant No. 2020YJZX0124).

## Conflict of Interest

The authors declare that the research was conducted in the absence of any commercial or financial relationships that could be construed as a potential conflict of interest.

## Publisher's Note

All claims expressed in this article are solely those of the authors and do not necessarily represent those of their affiliated organizations, or those of the publisher, the editors and the reviewers. Any product that may be evaluated in this article, or claim that may be made by its manufacturer, is not guaranteed or endorsed by the publisher.
